# *NRAS* and *KRAS* polymorphisms are not associated with hepatoblastoma susceptibility in Chinese children

**DOI:** 10.1186/s40164-019-0135-z

**Published:** 2019-05-09

**Authors:** Tianyou Yang, Yang Wen, Jiahao Li, Tianbao Tan, Jiliang Yang, Jing Pan, Chao Hu, Yuxiao Yao, Jiao Zhang, Yijuan Xin, Suhong Li, Huimin Xia, Jing He, Yan Zou

**Affiliations:** 1 Department of Pediatric Surgery, Guangzhou Women and Children’s Medical Center, Guangzhou Medical University, Guangzhou, Guangdong China; 2 First Affiliated Hospital of Sun Yat-Sen University, Guangzhou, Guangdong China; 3 Department of Pediatric Surgery, The First Affiliated Hospital of Zhengzhou University, Zhengzhou, Henan China; 4 Clinical Laboratory Medicine Center of PLA, Xijing Hospital, Air Force Medical University, Xi’an, Shaanxi China; 5 Department of Pathology, Children’s Hospital and Women’s Health Center of Shanxi, Taiyuan, Shannxi China

**Keywords:** Hepatoblastoma, Cancer susceptibility, *NRAS*, *KRAS*, Polymorphism

## Abstract

**Background:**

Hepatoblastoma is the most common hepatic malignancy in children, accounting for approximately 80% of all childhood liver tumors. *KRAS* and *NRAS*, members of the *RAS* gene family, are closely linked to tumorigenesis, and are frequently mutated in a variety of malignancies. They may thus play critical roles in tumorigenesis. However, there are few studies on the association between the *RAS* gene polymorphisms and risk of hepatoblastoma.

**Methods:**

We investigated whether the polymorphisms at these genes are associated with hepatoblastoma susceptibility in a hospital-based study of 213 affected Chinese children and 958 cancer-free controls. Genotypes were determined by TaqMan assay, and association with hepatoblastoma risk was assessed based on odds ratios and 95% confidence intervals.

**Results:**

No significant differences were observed between patients and controls in terms of age and gender frequency. All *NRAS* and *KRAS* genotypes are in Hardy–Weinberg equilibrium in the entire study population. We did not observe any significant association between hepatoblastoma risk and polymorphisms at *NRAS* and *KRAS*. The association between selected polymorphisms and hepatoblastoma risk was assessed after stratification by age, gender, and clinical stage. However, no significant association was observed even after stratification by age, gender, and clinical stage.

**Conclusions:**

The data suggest that *NRAS* and *KRAS* polymorphisms are irrelevant to hepatoblastoma susceptibility among Chinese population.

**Electronic supplementary material:**

The online version of this article (10.1186/s40164-019-0135-z) contains supplementary material, which is available to authorized users.

## Introduction

Hepatoblastoma, an embryonic tumor, accounts for about 80% of all childhood liver malignancies and 1% of all childhood malignancies [1, 2]. The most common clinical symptoms are abdominal masses usually accompanied by fever, weight loss, anorexia, obstructive jaundice, or acute abdominal bleeding due to tumor rupture [3, 4]. Of note, more than 90% of cases are associated with elevated levels of alpha-fetoprotein, an important biomarker [5].

Unlike adult hepatic cellular carcinoma, hepatoblastoma is not related to hepatitis B virus or liver cirrhosis [6]. Individual environmental risk factors may increase risk, while premature delivery and very low birth weight are associated with increased incidence [7]. The genetic disorders Beckwith–Wiedemann syndrome and familial adenomatous polyposis are closely associated with hepatoblastoma, suggesting that genetic factors may accelerate pathogenesis [2]. In addition, genetic polymorphisms that result in loss or alteration of the function of tumor-associated proteins may increase susceptibility to tumors and subsequent prognosis [8, 9]. Hence, genome-wide association studies of hepatoblastoma risk are warranted but rarely conducted.

The *RAS* genes *KRAS* and *NRAS* are believed to be closely linked to tumorigenesis [10]. *KRAS* is located on chromosome 12p12.1, and has diverse biological functions, including in angiogenesis, epidermal growth factor receptor (EGFR) signaling to the nucleus, and cell division, differentiation, proliferation, and growth [11–13]. Indeed, the RAS/RAF/MAPK pathway is one of the most important downstream pathways triggered by EGFR, and one that critically depends on *KRAS* and *NRAS* expression [14, 15]. The pathway is activated when an extracellular signaling molecule binds to and alters the conformation of a membrane receptor such as EGFR, which, in turn, binds to a series of proteins related to Ras activation, e.g., Grb2, SOS, etc. Ultimately, activated Ras triggers a phosphorylation cascade via MAPK to transduce the extracellular signal to the nucleus and elicit a response.

Mutations in *KRAS* and *NRAS* may constitutively activate signaling pathways downstream of EGFR, thereby promoting aberrant cell growth [16] and differentiation, which may then lead to tumorigenesis [17, 18]. Accordingly, patients with *KRAS* mutations do not respond to EGFR inhibitors [19]. Mutations in *KRAS* occur in about 30% to 40% of the population, and cluster at codons 12–13 of exon 2, and at codons 59, 61, and 17 of exon 3 [20, 21]. On the other hand, *NRAS* mutations are relatively uncommon, but result in malignant proliferation and metastasis [22]. Moreover, *NRAS* and *KRAS* mutations are much more common in the elderly [23].

*KRAS* and *NRAS* mutations are common in a variety of malignancies, including colorectal, pancreatic, and lung cancer [24, 25]. For example, such mutations are found in 20–50% and 1–6% of colorectal cancers, respectively [26]. Mutations in *KRAS* are also an early event in the development of pancreatic ductal adenocarcinoma, and are present in more than 90% of cases [27]. Further, *KRAS* mutations are found in about 22.5% to 36.0% of non-small cell lung cancers, of which about 97% are located in intron 12 and 13 [28]. On the other hand, *NRAS* mutations that are potentially targetable by therapy have been detected in small-cell lung cancer [29]. *RAS* mutations have also been detected in a small number of neuroblastoma patients. Of note, such mutations can be targeted effectively with everolimus, which is already on the market [30, 31]. Collectively, the growing body of evidence suggests that *RAS* mutations are present and may play important roles in a variety of solid tumors, including in the breast, cervix, small intestine, liver, and other organs [32]. Nevertheless, the relationship between *RAS* polymorphisms and hepatoblastoma has not been investigated. In this study, we analyzed the association between *NRAS* and *KRAS* polymorphisms and hepatoblastoma risk.

## Results

### Characteristics of the study population

The demographic characteristics of 213 hepatoblastoma patients and 958 controls recruited in Guangdong, Henan, Shaanxi, and Shannxi are listed in Additional file 1: Tables S1, S2. No significant differences were observed between patients and controls in terms of age and gender frequency, both as a single cohort or in each province.

### Association between hepatoblastoma risk and *NRAS* and *KRAS* polymorphisms

Genotypes at the *NRAS* polymorphism rs2273267 A > T are listed in Table 1 for hepatoblastoma patients and controls, along with those at the *KRAS* polymorphisms rs12587 G > T, rs7973450 A > G, and rs7312175 G > A. All *NRAS* and *KRAS* genotypes are in accordance with Hardy–Weinberg equilibrium in the entire study population. We did not observe any significant association between hepatoblastoma risk and polymorphisms at *NRAS* and *KRAS*. On the contrary, we found that subjects carrying the genotypes rs12587 TT, rs7973450 AG/GG, and rs7312175 GA/AA, alone or in combination, have a marginally lower risk of hepatoblastoma that is not statistically significant (adjusted odds ratio [OR] = 0.91; 95% confidence interval [CI] 0.67–1.25; *P* = 0.561), even though these genotypes are considered to indicate cancer risk.

**Table 1:** Association between hepatoblastoma risk and polymorphisms in *NRAS* and *KRAS*

Genotype	Patients (n = 213)	Controls (n = 958)	*P* ^a^	Crude OR (95% CI)	*P*	Adjusted OR (95% CI)^b^	*P* ^b^
*NRAS* rs2273267 A > T (HWE = 0.794)							
AA	103 (48.36)	486 (50.73)		1.00		1.00	
AT	88 (41.31)	395 (41.23)		1.05 (0.77–1.44)	0.755	1.05 (0.77–1.44)	0.758
TT	22 (10.33)	77 (8.04)		1.35 (0.80–2.27)	0.259	1.35 (0.80–2.27)	0.259
Additive			0.528	1.12 (0.89–1.40)	0.338	1.12 (0.89–1.40)	0.338
Dominant	110 (51.64)	472 (49.27)	0.531	1.10 (0.82–1.48)	0.531	1.10 (0.82–1.48)	0.532
Recessive	191 (89.67)	881 (91.96)	0.277	1.32 (0.80–2.17)	0.278	1.32 (0.80–2.17)	0.277
*KRAS* rs12587 G > T (HWE = 0.132)							
GG	128 (60.09)	609 (63.57)		1.00		1.00	
GT	79 (37.09)	300 (31.32)		1.25 (0.92–1.71)	0.158	1.26 (0.92–1.72)	0.155
TT	6 (2.82)	49 (5.11)		0.58 (0.24–1.39)	0.223	0.58 (0.24–1.39)	0.223
Additive			0.130	1.04 (0.80–1.34)	0.789	1.04 (0.80–1.34)	0.788
Dominant	85 (39.91)	349 (36.43)	0.342	1.16 (0.86–1.57)	0.342	1.16 (0.86–1.57)	0.341
Recessive	207 (97.18)	909 (94.89)	0.152	0.54 (0.23–1.27)	0.158	0.54 (0.23–1.27)	0.158
*KRAS* rs7973450 A > G (HWE = 0.213)							
AA	178 (83.57)	798 (83.30)		1.00		1.00	
AG	35 (16.43)	156 (16.28)		1.01 (0.67–1.50)	0.977	1.01 (0.67–1.50)	0.979
GG	0 (0.00)	4 (0.42)		/	/	/	/
Additive			0.640	0.95 (0.65–1.41)	0.814	0.95 (0.65–1.41)	0.811
Dominant	35 (16.43)	160 (16.70)	0.924	0.98 (0.66–1.46)	0.924	0.98 (0.66–1.46)	0.921
Recessive	213 (100.00)	954 (99.58)	0.345	/	/	/	/
*KRAS* rs7312175 G > A (HWE = 0.300)							
GG	167 (78.40)	740 (77.24)		1.00		1.00	
GA	44 (20.66)	200 (20.88)		0.98 (0.68–1.41)	0.892	0.98 (0.68–1.41)	0.892
AA	2 (0.94)	18 (1.88)		0.49 (0.11–2.14)	0.345	0.49 (0.11–2.15)	0.345
Additive			0.626	0.91 (0.65–1.26)	0.553	0.91 (0.65–1.26)	0.554
Dominant	46 (21.60)	218 (22.76)	0.714	0.94 (0.65–1.34)	0.714	0.94 (0.65–1.34)	0.715
Recessive	211 (99.06)	940 (98.12)	0.338	0.50 (0.11–2.15)	0.348	0.50 (0.11–2.15)	0.348
Combined effect of protective genotypes^c^							
0	139 (65.26)	605 (63.15)		1.00		1.00	
1	63 (29.58)	303 (31.63)		0.91 (0.65–1.26)	0.551	0.91 (0.65–1.26)	0.552
2	9 (4.23)	26 (2.71)		1.51 (0.69–3.29)	0.303	1.51 (0.69–3.29)	0.302
3	2 (0.94)	24 (2.51)		0.36 (0.09–1.55)	0.172	0.36 (0.09–1.55)	0.172
Trend			0.306	0.92 (0.73–1.16)	0.458	0.92 (0.73–1.16)	0.458
0	139 (65.26)	605 (63.15)		1.00		1.00	
1–3	74 (34.74)	353 (36.85)	0.564	0.91 (0.67–1.25)	0.564	0.91 (0.67–1.25)	0.561

*OR* odds ratio, *CI* confidence interval, *HWE* Hardy–Weinberg equilibrium

^a^By χ^2^ test vs. cancer-free controls

^b^Adjusted for age and gender

^c^Risk genotypes are rs12587 TT, rs7973450 AG/GG, and rs7312175 GA/AA

### Association of *NRAS* and *KRAS* polymorphisms with hepatoblastoma risk after demographic stratification

The association between select polymorphisms and hepatoblastoma risk was assessed after stratification by age, gender, and clinical stage (Tables 2, 3). However, no significant association was observed between hepatoblastoma risk and the *NRAS* rs2273267 A > T polymorphism in children aged more than 17 months (adjusted OR = 1.42, 95% CI 0.68–2.96, *P* = 0.350) or younger (adjusted OR = 1.23, 95% CI 0.62–2.43, *P* = 0.556, Table 2). Gender was also not linked to hepatoblastoma risk (adjusted OR = 1.84, 95% CI 0.90–3.77, and *P* = 0.094 for females, and adjusted OR = 0.97, 95% CI 0.47–1.97, and *P* = 0.925 for males). In addition, there was no significant correlation between stage I + II patients and the genotypes AA/AT and TT (adjusted OR = 1.77, 95% CI 0.94–3.32, *P* = 0.075), nor between such genotypes and stage III + IV patients (adjusted OR = 1.66, 95% CI 0.73–3.80, *P* = 0.229).

**Table 2:** Association between hepatoblastoma risk and *NRAS* rs2273267 A > T polymorphisms after stratification by age, gender, and clinical stages

Variables	rs2273267 (patients/controls)		Crude OR (95% CI)	*P*	Adjusted OR^a^ (95% CI)	*P* ^a^
	AA/AT	TT				
Age, months						
< 17	102/414	12/40	1.22 (0.62–2.41)	0.571	1.23 (0.62–2.43)	0.556
≥ 17	89/467	10/37	1.42 (0.68–2.96)	0.351	1.42 (0.68–2.96)	0.350
Gender						
Female	72/348	12/31	1.87 (0.92–3.82)	0.085	1.84 (0.90–3.77)	0.094
Male	119/533	10/46	0.97 (0.48–1.99)	0.942	0.97 (0.47–1.97)	0.925
Clinical stages						
I + II	84/881	13/77	1.77 (0.94–3.32)	0.075	1.77 (0.94–3.32)	0.075
III + IV	48/881	7/77	1.67 (0.73–3.81)	0.225	1.66 (0.73–3.80)	0.229

*OR* odds ratio, *CI* confidence interval

^a^Adjusted for age and gender, with the stratification factor omitted

**Table 3:** Association between *KRAS* genotypes and hepatoblastoma susceptibility after stratification by age, gender, and clinical stages

Variables	rs12587 (patients/controls)		AOR (95% CI)^a^	*P* ^a^	rs7973450 (patients/controls)		AOR (95% CI)^a^	*P* ^a^	rs7312175 (patients/controls)		AOR (95% CI)^a^	*P* ^a^	Combined genotypes (patients/controls)		AOR (95% CI)^a^	*P* ^a^
	GG	GT/TT			AA	AG/GG			GG	GA/AA			0	1–3		
Age, months																
< 17	70/278	44/176	1.00 (0.66–1.53)	0.998	90/371	24/83	1.20 (0.72–2.00)	0.486	94/358	20/106	0.70 (0.41–1.19)	0.189	73/275	41/179	0.87 (0.57–1.33)	0.517
≥ 17	58/331	41/173	1.35 (0.87–2.10)	0.179	88/427	11/77	0.69 (0.35–1.36)	0.286	73/392	26/112	1.25 (0.76–2.04)	0.383	66/330	33/174	0.95 (0.60–1.50)	0.817
Gender																
Female	49/222	35/157	0.99 (0.61–1.60)	0.963	68/311	16/68	1.03 (0.56–1.90)	0.916	67/284	17/95	0.76 (0.42–1.35)	0.344	54/223	30/156	0.77 (0.47–1.26)	0.302
Male	79/387	50/192	1.27 (0.86–1.89)	0.231	110/487	19/92	0.92 (0.54–1.57)	0.750	100/456	29/123	1.07 (0.68–1.70)	0.765	85/382	44/197	1.01 (0.67–1.50)	0.980
Clinical stages																
I + II	56/609	41/349	1.28 (0.84–1.97)	0.249	75/798	22/160	1.47 (0.89–2.44)	0.132	76/740	21/218	0.94 (0.57–1.56)	0.803	60/605	37/353	1.06 (0.69–1.64)	0.784
III + IV	32/609	23/349	1.25 (0.72–2.17)	0.430	48/798	7/160	0.73 (0.32–1.64)	0.445	44/740	11/218	0.85 (0.43–1.67)	0.626	37/605	18/353	0.83 (0.47–1.48)	0.532

*AOR* adjusted odds ratio, *CI* confidence interval

^a^Adjusted for age and gender, with the stratification factor omitted

Further analysis also showed that hepatoblastoma risk was not significantly associated with the *KRAS* polymorphisms rs12587 G > T, rs7973450 A > G, and rs7312175 G > A in children aged more than 17 months (*P* = 0.179, *P* = 0.286, and *P* = 0.383) or younger (*P* = 0.998, *P* = 0.486, and *P* = 0.189), nor in females (*P* = 0.963, *P* = 0.916, and *P* = 0.344) and males (*P* = 0.231, *P* = 0.750, and *P* = 0.765). There was also no significant correlation between stage I + II patients and the genotypes AA/AT and TT (adjusted OR = 1.06, 95% CI 0.69–1.64, *P* = 0.784), nor between such genotypes and stage III + IV patients (adjusted OR = 0.83, 95% CI 0.47–1.48, *P* = 0.532).

## Discussion

Hepatoblastoma is a rare pediatric embryonic tumor with incidence of about 1/1,000,000 [33], and is often associated with chromosomal abnormalities, especially at chromosome 2, 11, 18, and 20 [34]. However, the relative risk of hepatoblastoma is 2280 times higher in children with Beckwith–Wiedemann syndrome, indicating that aberrations in chromosome 11 play an important role in pathogenesis [35]. Similarly, the risk is 1220-fold higher in children with familial adenomatous polyposis, implying that lesions in chromosome 5 are also involved [36]. Of note, somatic mutation of the tumor suppressor *APC*, which is located on chromosome 5, is present in 67–89% of sporadic hepatoblastoma. Such mutations occur at the 5′ half of the gene, and generally considered to be at or near base pair 1309 [37]. Finally, some genes that are typically imprinted and differentially methylated are already abnormally methylated even before the development of hepatoblastoma, suggesting that methylation at these sites is related to pathogenesis [38].

RAS is a membrane-bound GTP/GDP-binding protein and an important proto-oncogene in intracellular EGFR signaling [39]. Accordingly, it is an essential regulator of cell proliferation and angiogenesis, and regarded as a molecular switch that senses and transmits extracellular stimuli of proliferation, growth, differentiation, and related processes [40]. Indeed, *RAS* genes, including *KRAS*, *HRAS*, and *NRAS*, are all implicated in tumorigenesis. For example, activating mutations in *RAS* may cause continuous growth, dedifferentiation of cells, and tumor development [41].

Currently, the relationship between *KRAS* mutations and clinical outcomes is not fully elucidated. On one hand, Chang et al. [42] found that *KRAS* mutations are associated with tumor size, degree of differentiation, lymph node metastasis, and poor prognosis. Similarly, Zhang et al. [43] found that *KRAS* mutations were significantly more frequent in Chinese patients with mucinous colorectal adenocarcinomas and well-differentiated colorectal cancers, implying that *KRAS* mutations in such patients are causative but different from those patients in Western countries. Our data also show that hepatoblastoma risk in Chinese patients is not significantly associated with polymorphisms in *NRAS* and *KRAS*, even after stratification by age, gender, and clinical stage.

We note that although synergistic interactions between environmental and genetic factors contribute to the development of hepatoblastoma, we did not collect data on parental exposure to hazards, diets, and lifestyles. In addition, our cohort is certainly not representative of the whole Chinese population. Nevertheless, the findings are probably not generalizable to other races. Finally, the sample size is relatively small, and thus has limited statistical power. These issues should be avoided as much as possible in future studies to better investigate the relationship between hepatoblastoma risk and *NRAS* and *KRAS* polymorphisms.

## Conclusions

We find that *NRAS* and *KRAS* polymorphisms are irrelevant to hepatoblastoma susceptibility among Chinese population. Moreover, further investigations of polymorphisms that might mediate the risk of hepatoblastoma would help gain a better understanding of the pathogenesis and improve prognosis.

## Materials and methods

### Study population

The cohort consisted of 213 hepatoblastoma cases diagnosed by histopathology in Guangdong, Henan, Shaanxi, and Shanxi. There are no direct blood relationships among cases, and 958 cancer-free children were included as controls (Additional file 1: Table S1). Written informed consent was obtained from legal guardians, and the protocol was approved by the institutional review board at Guangzhou Women’s and Children’s Medical Center.

### DNA extraction and genotyping

*NRAS* and *KRAS* polymorphisms were genotyped in blinded fashion using TaqMan real-time PCR [44–47]. Assays were repeated for 10% of randomly selected samples, and results were 100% concordant with original genotypes.

### Statistical analysis

The demographic characteristics of and genotype frequency distribution in cases and controls were compared by χ^2^ test. Deviation from Hardy–Weinberg equilibrium was tested in control subjects using χ^2^ goodness-of-fit test. Odds ratios and 95% confidence intervals were calculated to assess the association between hepatoblastoma risk and *NRAS* and *KRAS* polymorphisms. Age, gender, and clinical stages were compared by χ^2^ test and logistic regression among patients with different genotypes. Polymorphic loci were evaluated using dominant, recessive, and additive models, and corresponding *P* values, relative risk odds ratios, and 95% confidence intervals were calculated. All statistical analyses were performed in SAS version 9.4 (SAS Institute, Cary, NC), with *P* values < 0.05 considered as statistically significant.

## Additional file

**Additional file 1: Table S1.** Frequency distribution of select variables in hepatoblastoma patients and cancer-free controls. **Table S2.** Demographic characteristics of the study population.
