# Supplementary material for: NRAS and KRAS polymorphisms are not associated with hepatoblastoma susceptibility in Chinese children
Source: Exp Hematol Oncol. 2019 May 9;8:11. doi: 10.1186/s40164-019-0135-z (PMC6507155; doi:10.1186/s40164-019-0135-z)
Supplement: Supplementary file 1 — Additional file 1: Table S1. Frequency distribution of select variables in hepatoblastoma patients and cancer-free controls. Table S2. Demographic characteristics of the study population. [file 40164_2019_135_MOESM1_ESM.docx]

| **Table S1 \|** Frequency distribution of select variables in hepatoblastoma patients and cancer-free controls. | | | | | | | | | | | | |
| --- | --- | --- | --- | --- | --- | --- | --- | --- | --- | --- | --- | --- |
| Variables | Guangdong province | | | Henan province | | | Shaanxi province | | | Shannxi province | | |
|  | Patients  (n = 146) | Controls  (n = 438) | *P*^a^ | Patients  (n = 42) | Controls  (n = 176) | *P*^a^ | Patients  (n = 15) | Controls  (n = 186) | *P*^a^ | Patients  (n = 10) | Controls  (n = 158) | *P*^a^ |
|  | No. (%) | No. (%) |  | No. (%) | No. (%) |  | No. (%) | No. (%) |  | No. (%) | No. (%) |  |
| Age range, months | 0.63-149.97 | 0.07-156.00 | 0.214 | 0.83-108.00 | 0.10-108.00 | 0.285 | 3.60-72.00 | 0.03-60.00 | 0.286 | 0.23-72.00 | 0.004-60.00 | 0.785 |
| Mean age ± SD | 23.16 ± 24.59 | 23.11 ± 18.62 |  | 26.73 ± 24.96 | 27.28 ± 18.87 |  | 21.50 ± 24.20 | 23.66 ± 16.66 |  | 20.32 ± 20.38 | 21.70 ± 18.28 |  |
| <17 | 79 (54.11) | 211 (48.17) |  | 21 (50.00) | 72 (40.91) |  | 9 (60.00) | 85 (45.70) |  | 5 (50.00) | 86 (54.43) |  |
| ≥17 | 67 (45.89) | 227 (51.83) |  | 21 (50.00) | 104 (59.09) |  | 6 (40.00) | 101 (54.30) |  | 5 (50.00) | 72 (45.57) |  |
| Gender |  |  | 0.961 |  |  | 0.830 |  |  | 0.544 |  |  | 0.912 |
| Female | 58 (39.73) | 175 (39.95) |  | 15 (35.71) | 66 (37.50) |  | 7 (46.67) | 72 (38.71) |  | 4 (40.00) | 66 (41.77) |  |
| Male | 88 (60.27) | 263 (60.05) |  | 27 (64.29) | 110 (62.50) |  | 8 (53.33) | 114 (61.29) |  | 6 (60.00) | 92 (58.23) |  |
| Clinical stages |  |  |  |  |  |  |  |  |  |  |  |  |
| I | 6 (4.11) | / |  | 19 (45.24) | / |  | 15 (100.00) | / |  | 2 (20.00) | / |  |
| II | 46 (31.51) | / |  | 3 (7.14) | / |  | / | / |  | 6 (60.00) | / |  |
| III | 37 (35.34) | / |  | 3 (7.14) | / |  | / | / |  | 0 (0.00) | / |  |
| IV | 12 (8.22) | / |  | 1 (2.38) | / |  | / | / |  | 2 (20.00) | / |  |
| NA | 45 (30.82) | / |  | 16 (38.10) | / |  | / | / |  | / | / |  |
| SD, standard deviation.  ^a^ By two-sided χ^2^ test comparing hepatoblastoma patients and cancer-free controls. | | | | | | | | | | | | |

| **Table S2 \|** Demographic characteristics of the study population. | | | | | |
| --- | --- | --- | --- | --- | --- |
| Variables | Patients (n = 213) | | Controls (n = 958) | | *P*^a^ |
|  | No. | % | No. | % |  |
| Age range, months | 0.23-149.97 | | 0.004-156.00 | | 0.105 |
| Mean age ± SD, months | 23.62 ± 24.36 | | 23.75 ± 18.30 | |  |
| < 17 months | 114 | 53.52 | 454 | 47.39 |  |
| ≥ 17 months | 99 | 46.48 | 504 | 52.61 |  |
| Gender |  |  |  |  | 0.973 |
| Female | 84 | 39.44 | 379 | 39.56 |  |
| Male | 129 | 60.56 | 579 | 60.44 |  |
| Clinical stage |  |  |  |  |  |
| I | 42 | 19.72 |  |  |  |
| II | 55 | 25.82 |  |  |  |
| III | 40 | 18.78 |  |  |  |
| IV | 15 | 7.04 |  |  |  |
| NA | 61 | 28.64 |  |  |  |
| ^a^ By two-sided χ^2^ test vs. controls. | | | | | |
